# Home Monitoring for the Management of Age-Related Macular Degeneration: A Review of the Development and Implementation of Digital Health Solutions over a 25-Year Scientific Journey

**DOI:** 10.3390/medicina61122193

**Published:** 2025-12-11

**Authors:** Miguel A. Busquets, Richard A. Garfinkel, Deepak Sambhara, Nishant Mohan, Kester Nahen, Gidi Benyamini, Anat Loewenstein

**Affiliations:** 1 Retina Associates of Kentucky, Lexington, KY 40509, USA; mabbusquets@gmail.com; 2 Retina Group of Washington, Chevy Chase, MD 20815, USA; ragarfinkel@gmail.com; 3 Eye Clinic of Wisconsin, Wausau, WI 54403, USA; deepaksambhara@gmail.com; 4 Notal Vision Inc., Manassas, VA 20109, USA; nmohan@notalvision.com (N.M.); kester.nahen@notalvision.com (K.N.); gidi@notalvision.com (G.B.); 5 Tel Aviv Medical Center, Tel Aviv 6423906, Israel

**Keywords:** AMD, neovascular age-related macular degeneration, preferential hyperacuity perimetry, Artificial Intelligence, OCT, home OCT, telemedicine, digital healthcare provider

## Abstract

The management of age-related macular degeneration (AMD) presents a significant challenge attributable to high disease heterogeneity. Patient realization of symptoms is poor and it is urgent to treat before permanent anatomic damage results in vision loss. This is true for the initial conversion from non-exudative intermediate AMD (iAMD) to exudative AMD (nAMD), and for the recurrence of nAMD undergoing treatment. Starting from the essential requirements that any practical solution needs to fulfill, we will reflect on how persistent navigation towards innovative solutions during a 25-year journey yielded significant advances towards improvements in personalized care. An early insight was that the acute nature of AMD progression requires frequent monitoring and therefore diagnostic testing should be performed at the patient’s home. Four key requirements were identified: (1) A tele-connected home device with acceptable diagnostic performance and a supportive patient user interface, both hardware and software. (2) Automated analytics capabilities that can process large volumes of data. (3) Efficient remote patient engagement and support through a digital healthcare provider. (4) A low-cost medical system that enables digital healthcare delivery through appropriate compensation for both the monitoring provider and the prescribing physician services. We reviewed the published literature accompanying first the development of Preferential Hyperacuity Perimetry (PHP) for monitoring iAMD, followed by Spectral Domain Optical Coherence Tomography (SD-OCT) for monitoring nAMD. Emphasis was given to the review of the validation of the core technologies, the regulatory process, and real-world studies, and how they led to the release of commercial services that are covered by Medicare in the USA. We concluded that while during the first quarter of the 21st century, the two main pillars of management of AMD were anti-VEGF intravitreal injections and in-office OCT, the addition of home-monitoring-based digital health services can become the third pillar.

## 1. Introduction

The management of age-related macular degeneration (AMD) presents a significant challenge attributable to high disease heterogeneity—both with regard to the time of conversion from intermediate to advanced stages and to the response to treatment [1,2]. Patient realization of symptoms is poor, and the urgency to treat AMD before permanent anatomic damage results in vision loss is high. This is true for the initial conversion from non-exudative intermediate AMD (iAMD) to exudative AMD (nAMD) [3,4,5], and for the recurrence of nAMD undergoing treatment [6].

Therefore, timely monitoring and diagnosis as well as personalized intervention is a long-standing challenge for eyes diagnosed with both iAMD and nAMD [7,8]. These challenges exist in routine clinical management and clinical studies towards the development of new therapies. Starting from the essential requirements that any practical solution needs to fulfill, we will reflect on how persistent navigation towards innovative solutions during a 25-year journey yielded significant advances towards improvements in personalized care.

An early insight into the development of new patient-centric solutions was that the acute nature of AMD progression requires frequent monitoring, and therefore diagnostic testing should be performed at the patient’s home. In general, home diagnostic solutions for monitoring AMD should meet four essential requirements:

1. Acceptable system performance addressing clinical needs, including a user interface, both hardware ergonomics and a software application, and a tele-connected device that can be self-installed and self-operated by the target population with a chronic retinal disease in a home environment.
2. Automated analytics capabilities that can process large volumes of data from near-daily patient monitoring in support of a prescribing physician’s review and utilization of the data.
3. Efficient remote patient engagement and support through a physician supervised digital healthcare provider entity. Such an entity should maintain ongoing relationships with a large number of patients under the care of a large number of clinics and physicians who prescribe the monitoring program.
4. Low-cost medical device and data management solutions that can justify the use by a single patient, and a digital healthcare business model that allows proper compensation for both the technical services of the monitoring provider and the professional services of the prescribing physician.

This review of published literature will focus on solutions that a team of engineers and collaborating clinicians have brought from the bench to patients’ homes.

## 2. Dry AMD

The journey to develop personalized diagnostic solutions, the subject of this report, started in the year 2000. At that time, the first anti-VEGF treatments were years from approval, and no effective therapy was available for iAMD and most cases of nAMD. The initial decision to develop a solution for the more stable part of the disease process, iAMD, was therefore the most clinically meaningful.

In the absence of an existing solution that can be adopted to meet the four requirements, a proprietary core technology was developed for iAMD monitoring, termed Preferential Hyperacuity Perimetry (PHP). It was specifically designed to detect visual field disturbances associated with nAMD prior to overt visual symptoms. This technology is based on hyperacuity (known also as vernier acuity)—the ability to perceive a very small difference in the relative spatial localization of two or more visual stimuli—that can be locally impaired before visual acuity is affected [9,10]. Early nAMD causes subtle separation of the different layers of the retina, resulting in localized distortion of vision (metamorphopsia), often with accompanying loss of sensitivity (scotoma). The PHP test is applied to the central area of the visual field of 14°. Based on a comparison of the test results with a normative database and with the participant’s baseline performance with the PHP device, the system determines whether there are visual responses which may be associated with progression from iAMD to nAMD [11].

The same core technology was implemented in several products branded as Macular Computerized Psychophysical Test (MCPT) [12], Preview PHP [13], and Foresee PHP, all for office use, and later in the ForeseeHome for home use.

The first iteration of the PHP solution for early detection of the first conversion was developed for a supervised office setting. Such a system did not have to meet all four requirements, and it enabled a shorter time to market. The performance of the system were validated in several trials that allowed it to receive clearance from the US Food and Drug Administration (FDA) and to be launched commercially [13,14,15,16,17,18]. The system was also included in studies of other diseases where progression typically causes visual distortions, including hydroxychloroquine retinal toxicity, Best vitelliform macular dystrophy, idiopathic epiretinal membrane, and neovascular AMD [19,20,21,22,23].

During the early 2000s, the Zeiss Stratus time domain (TD) OCT (Carl Zeiss Meditech, Dublin, CA, USA) transformed from being a limited research tool to a practical clinical tool and emerged as an important diagnostics solution. The strengthening of the set of in-office diagnostics tools with the TD-OCT reinforced the motivation to stay the course of the original plan of meeting all four requirements with a home solution for iAMD. A solution branded ForeseeHome (FSH) was developed. It is a self-installed and operated small form factor PHP device intended for eyes diagnosed with iAMD with visual acuity of 20/60 or better, with built-in telecommunication capabilities [24].

The system includes the core PHP technology, a low-cost self-operated device, and an automated analysis of the testing results. It utilizes a dedicated monitoring center operated as an accredited Independent Diagnostic Testing Facility (IDTF) infrastructure, customer management systems, and dedicated Current Procedural Terminology (CPT) codes that support Medicare reimbursement (0379T).

The FSH system satisfies all four requirements. Of particular interest is the early implementation of an AI machine learning algorithm that calculates a test score from the responses of the patient to the presented hyperacuity signals.

For broader context of the landscape, a study of three selected home monitoring solutions for eyes diagnosed with iAMD in the UK included the KeepSight Journal (KSJ, American Macular Degeneration Foundation, Northampton, MA, USA), the MyVisionTrack v1.3.1 (mVT, F. Hoffmann-La Roche Ltd., Basel, Switzerland) vision-monitoring mobile app, and the MultiBit (MBT, Visumetrics AB, Göteborg, Sweden) app. The results of the study suggest that none of these home-monitoring vision tests provided satisfactory diagnostic accuracy to identify active nAMD [25].

There are several reasons why early detection of conversion of eyes diagnosed with iAMD is a difficult problem to solve. Among them is the ability of the visual system of the human brain to compensate for any distortions and areas of scotoma for a long period after anatomical changes started to take place. The limitation in self-identification of early changes may be explained by the mechanisms of scanning, completion and filling-in, and integration of information from a fellow eye, as well as by varying environmental conditions impacting test performance. This is possibly the reason why until now, competitive technologies, including the Amsler grid, while continuing to have a clinical role in patients’ self-assessment as a universally accessible option, and the above-mentioned home monitoring solutions, demonstrated inferior performance or failed to ever report proper efficacy compared to FSH [26,27,28,29,30].

As with the office version of the PHP, FSH was initially validated in a cross-sectional study. The study established that the FSH test can distinguish eyes with iAMD from eyes that recently converted to nAMD with high sensitivity and specificity as compared to a gold standard of fluoresceine angiography (FA). The validation of the performance of a single test was the cornerstone of any effort to develop a longitudinal, at-home solution [31].

The journey then continued to at-home longitudinal studies, with the foremost being the HOME study which was ancillary to the AREDS2 study [32]. The study screened 1970 patients to enroll 1520 of them. Randomization divided the participants into groups of 763 and 757 to use FSH or be included in a control arm, respectively. It was likely one of the largest diagnostic device randomized controlled trials in the field of Ophthalmology. The study was stopped early by the Data Safety and Monitoring Committee in a pre-planned interim analysis after showing that monitoring with FSH resulted in earlier detection of conversion from iAMD to nAMD in terms of change from baseline visual acuity. Four reports that reviewed the study design, the main findings, the effectiveness of the implementation of the system, and the detailed analysis of the findings of the reading center were published by the study investigators. These publications paved the way to having the service paid for by Medicare, materializing one of the key requirements for viability [11,33,34,35].

Further publications provided additional perspectives and validation of the performance of the system [36,37]. Since the launch of FSH, as of October 2025, over 50,000 patients used the system to protect their vision, performing over 17 million tests. Ongoing data collection allowed the performance of the system to continue to be monitored in real-world settings. The two largest retrospective analyses of up to 10 years of real-world data reported that 306 and 285 conversions were detected by the FSH strategy that combines FSH alerts, patient-reported symptoms, and office visits. At conversion, 81% (95% CI 72–88%) and 84% (95% CI 78–88%) of the eyes in the two analyzed cohorts maintained visual acuity of 20/40 or better, respectively. The two cohorts included 8991 and 2123 patients that reported on the usage of the FSH system for 11,525 and 10,474 monitoring years with a high and persistent testing pattern of a mean (SD) of 5.6 (3.2) and 5.2 (3.4) tests per week, respectively.

These are positive key performance indicators for the work of the remote monitoring and patient support center, one of the four elements of the system [38,39]. These findings are consistent with those reported in the HOME study. They can be attributed to the fact that the exact same test and analysis with the exact same device, controlling the test conditions including the screen contrast and resolution, distance, ambient light and customer support infrastructure, were used across all cohorts.

Such a remarkable translation from a randomized controlled trial to real-world clinical care is not often seen in emerging technologies. It can be concluded that although the observed patients’ adherence to near-daily monitoring was high, to ensure sustained patient engagement over many years, as the users become older and may suffer from co-morbidities, ongoing engagement and feedback by a dedicated monitoring and support center are essential.

A cost-effectiveness analysis based on the HOME study results concluded that home telemonitoring of patients with iAMD who are at risk for nAMD was cost-effective compared with scheduled examinations alone [40].

The large database of real-world data also allowed analysis of the eventual outcomes of eyes that received FSH alerts; however, nAMD was not identified during a prompt office visit. A Kaplan–Meier survival analysis showed that these eyes are at elevated risk for a future conversion. The risk level varies by the status of the fellow eye of iAMD vs. nAMD from 10% to 43% in 2 years, respectively. Eyes with non-exudative alerts should be followed closely and are good candidates to study prophylactic treatments of iAMD [41].

Based on published clinical evidence, the 2024 American Academy of Ophthalmology Preferred Practice Pattern guidelines elevated the FSH monitoring program to be comparable to the Amsler grid, which has long been considered the gold standard for patient self-assessment aiding early detection of advanced AMD [42].

## 3. Wet AMD

The unmet need in the management of wet AMD relates to obtaining prompt information about disease reactivation requiring treatment, and a better understanding of treatment response.

Since the time that effective anti-VEGF treatments were introduced, Spectral Domain (SD) OCT, now available from several vendors, has become the standard tool for the management of eyes with AMD [43]. The excellent imaging capabilities of SD-OCT, specifically as it relates to the visualization of hypo-reflective spaces (HRS) associated with retinal fluid, created a perfect match with anti-VEGF therapy, which is primarily aimed at resolving retinal fluid [44].

The addition of aflibercept to the existing arsenal of bevacizumab and ranibizumab supported longer extension periods, even beyond the on-label 8-week intervals, and the Treat and Extend (T&E) approach of treatment administration at every visit and use of OCT finding to determine the timing of the subsequent visit became the most common treatment paradigm in the US [45,46]. The ability to extend treatment intervals has received a further boost by the approval of longer acting therapies including faricimab and aflibercept 8 mg, and the pipeline of several novel mechanisms aiming to achieve this goal. The heterogeneous response to these treatments has highlighted the need for home monitoring to better personalize retreatment intervals, reduce the burden on patients, and provide a safety net.

These advancements of therapeutic solutions and the introduction of structural OCT-based biomarkers for disease management motivated the development of the first self-installed and self-operated home OCT device. In contrast to monitoring iAMD, in which a core testing technology had to be developed from scratch, the main core diagnostics effort for nAMD was adaptive in nature, and the major innovations were related to form factors and user interaction that support home use at low cost.

In support of these requirements, including device cost consideration, a scan field of 3 mm × 3 mm was selected, representing a widely acceptable clinical requirement to visualize the key part of the macula surrounding the fovea. Other ergonomic design features support the usability of the system, and specifically, the ability of the patients to position their pupil in front of the device at the correct distance and lateral position. A proprietary positioning and fixation guidance module was developed to allow self-imaging without the presence of a technician, in contrast to in-office OCT, resulting in a design that can be affordable for a single user. The home OCT system utilizes the same cloud infrastructure as ForeseeHome, and the same patient support services that are essential to monitor adherence and provide feedback to the user to ensure long term compliance with the required frequency of self-imaging.

A parallel effort took place to develop automated image analysis capabilities for large volumes of SD-OCT scans. The Notal OCT Analyzer (NOA) AI application was developed first to process Cirrus HD OCT (Carl Zeiss Meditech, Dublin, CA, USA) scans.

A study comparing the NOA output to expert retina specialist grading in the identification of retinal fluid and ranking of B-scans by the amount of fluid was conducted. The results were published in 2016 and are probably the first to be reported in the field, which was later followed by many other groups and led to the founding of several companies [47].

NOA was also adopted to analyze Spectralis images (Heidelberg Engineering, Heidelberg, Germany), including further validation of its performance [48], that was followed by several additional publications with growing numbers of images.

Of special interest are the analysis of a UK real-world dataset that reported the relationship between fluid fluctuations and vision loss; a report of real-world findings from a very large bi-national dataset of ~80,000 images that was included in the BIRAX project; Age-Related Eye Disease Study 2 10-year Follow-On Study (AREDS2-10Y) that showed that AI performed superior to retina specialists in identification of fluid; and an analysis of the fluid volume of 521 eyes in the Truckee study that reported sustained reduction in retinal fluid volume after switching to faricimab [49,50,51,52].

An important milestone in the evolution of analytical tools for OCT was the publication of an American Journal of Ophthalmology (AJO) perspective by experts in the field that compared results from several studies and several algorithms and concluded with a recommendation that the volume unit of a nano-liter is preferred as the standard for reporting retinal HRS typically associated with fluid. This recommendation was de facto accepted by many of the developers of such algorithms and advanced our field towards consistent reporting of a new meaningful biomarker of disease activity and treatment response [53].

In order to adopt the NOA to analyze the images of home OCT, cross-sectional studies were performed with the purpose of creating a learning dataset for the algorithm. Following manual annotation by graders and a deep learning process, a NOA version for home OCT was released.

As with the office PHP and FSH, the first cornerstone validation step for the home OCT device was performed with a large cross-sectional study that enrolled 600 participants’ eyes. It showed that the target population with visual acuity of 20/320 or better, as was eventually cleared by the FDA, can self-image and produce high quality volume scans that can be analyzed by NOA with a high level of repeatability [54].

The successful results of the cross-sectional study led to a series of longitudinal studies. The first was a pilot of 4 patients who were monitored for 4 weeks; it was the first ever at-home OCT study, marking a modest, yet significant milestone [55]. Additional validation involved three studies performed in the USA.

The first one enrolled 15 patients for three-month follow-up. The clinical management of their nAMD did not consider the collected home OCT data, and the patients maintained their standard of care plan. The study dataset included 2380 self-images allowed for comparison with in-office OCT [56]. The study data was analyzed independently by a group from Roche Pharma Research and Early Development Pharmaceutical Sciences, Roche Innovation Center Basel, F. Hoffmann-La Roche Ltd., Basel, Switzerland, that investigated if home OCT combined with pharmacokinetic/pharmacodynamic (PK/PD) modeling can reduce the required sample size in early trials for retinal diseases (e.g., nAMD). The analysis showed that for a given desired effect, home OCT monitoring allowed a 20–40% sample size reduction when compared to traditional biweekly in-clinic monitoring. These findings highlight the potential for home OCT and PK/PD modeling to improve trial efficiency and patient convenience while maintaining statistical power which can streamline clinical trials, expedite the development of new retinal therapies, and improve patient access to treatment [57].

The second study involved 15 patients and a 6-month follow-up—DRCR Retina Network Protocol AK. The study enrolled naïve nAMD eyes and served as a pilot to the DRCR Protocol AO randomized controlled trial. During the study, the investigators were not exposed to the home OCT data. The study demonstrated the wide variability in retinal HRS dynamics patterns of naïve eyes and reinforced the need for personalized monitoring and treatments [58].

Following the success of DRCR Protocol AK, DRCR Protocol AO was initiated. It is a large randomized controlled trial with an anticipated enrollment of 600 participants randomized to compare real-world Treat and Extend and home OCT-based management of naïve nAMD eyes using faricimab. The results are expected in 2028 [59].

The data collected from these at-home studies allowed an evaluation of the impact that HRS volume trajectories have on clinical decision-making. This includes the timing of treatment as well as the type of drug used. Fifteen retina specialists reviewed 10 trajectories each. Home OCT-based decisions differed substantially from actual standard clinical care in more than 40% of the cases, demonstrating that home OCT has the potential to facilitate more personalized care in nAMD [60].

The third study was the first prospective clinical trial in which the physicians used home OCT data to manage the disease and make treatment decisions [59]. It included 15 patients and a 6-month follow-up. The physician involvement entailed setting up HRS volume and time-based notification thresholds that when reached, triggered a reminder to access the report, enabling decisions about the need for an office visit. It enrolled pre-treated eyes and showed that home OCT allowed maintaining the patients’ vision while reducing the treatment burden by 48% compared to the same eyes’ preceding reference period [61].

This study enabled the publication of two interesting cases. One case of neovascular type 3, as defined by the Consensus Nomenclature for Reporting Neovascular AMD Data (CONAN), concluded that home OCT guided treatment decisions may serve as a useful management framework with significant potential for optimization [62], and in a second case of an eye with a Susvimo implant (Genentech, South San Francisco, CA, USA), refill decisions were driven by review of the home OCT HRS trajectory. It demonstrated a new treatment paradigm combining sustained drug delivery and remote monitoring. The combination of sustained drug delivery and remote monitoring has the potential to reduce the total patient treatment burden while optimizing outcomes [63].

In the absence of a predicate device cleared by the FDA, the regulatory path to achieve marketing authorization for home OCT required a de novo process. Two pivotal trials enrolled over 500 patients.

The first study evaluated the usability and performance of the system in the visualization of retinal HRS—typically representing fluid, as compared to a reference in-office OCT in a five-weeks-at-home longitudinal study. The second study evaluated the performance of NOA in the segmentation of HRS as compared to reading center graders [64,65].

An additional analysis provided a longitudinal validation of the NOA for the identification of clinically significant changes in the trajectories of total retinal HRS by applying NOA to analyze the data collected in the longitudinal pivotal study. A personalized approach based on the reference change value (RCV) methodology used in laboratory medicine comprised HRS volume curve-fitting to evaluate the change in amplitude (signal) and within-subject variations (noise). It was followed by receiver operating characteristic (ROC) analysis of the signal-to-noise ratio (SNR) to identify the optimal threshold for determination of a clinically significant change in HRS. NOA and its trajectories were found to be a reliable tool for identifying clinically relevant changes in retinal HRS status by the quality standards of laboratory medicine [66].

**Figure 1:**
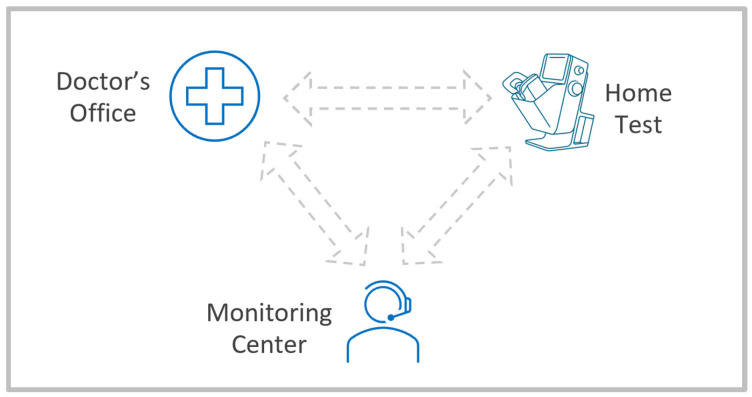
An illustration of the digital healthcare provider model.

The digital healthcare provider model that is implemented for monitoring services of iAMD and nAMD is illustrated in Figure 1.

To enable compensation of the monitoring center and prescribing physician for the unique technical and professional services involved in home OCT monitoring, dedicated CPT codes are required. Following a digital healthcare provider nominee’s request that was supported by the American Academy of Ophthalmology and the American Society of Retina Specialists, the American Medical Association’s CPT Panel established a group of new technology codes for remote OCT (0604T, 0605T, 0606T) in 2020.

As a result of the marketing authorization by the FDA, the Center for Medicare and Medicaid Services (CMS) established national pricing for 0605T supporting the cost of service, hence meeting the fourth requirement to bring a new remote patient monitoring program to patients in need.

## 4. Future Directions for Home Monitoring

The validated performance of the first home OCT ignited interest in monitoring additional diseases, specifically those that are associated with exudation.

Home OCT was studied for monitoring Diabetic Macular Edema (DME). Thirteen participants of the phase 1 THAMES study (NCT06850922) were monitored with home OCT for 6 months. The end point for the study was Central Subfield Thickness (CST), and the study concluded that the measurements of home OCT correlated strongly with office-based OCT, and that home OCT may play a potential role for self-imaging in the assessment of DME disease activity and the monitoring of treatment response [67].

A study for monitoring eyes diagnosed with central serous chorioretinopathy (CSCR) is currently ongoing. Additional possible applications may include monitoring eyes diagnosed with retinal vein occlusions and monitoring iAMD for progression to geographic atrophy or conversion to nAMD.

## 5. Conclusions

We reviewed a 25-year journey of the development of remote patient monitoring solutions to address the unmet needs of the growing population of patients diagnosed with AMD and other retinal diseases.

It began with a clear set of four independent requirements that guided the development of hardware, software, patient user interaction, analytics tools, and clinical implementation. Throughout the years, rigorous evaluations in clinical studies and real-world practice were performed and reported in peer reviewed publications. This process prompted feedback and generated evidence towards establishing digital health services as alternatives to subjective patient self-assessments or frequent cumbersome office appointments.

Our review provides a broad overview of the extensive efforts that were required to make remote patient monitoring a reality for physicians and their patients suffering from sight-threatening retinal diseases.

In the first quarter of the 21st century, the two main pillars of management of AMD were anti-VEGF intravitreal injections [68] and OCT [43]. We look forward to the addition of home-monitoring-based digital health services as the third pillar of patient-centric personalized healthcare.

## Data Availability

No new data were created or analyzed in this study. Data sharing is not applicable to this article.

## Abbreviations

The following abbreviations are used in this manuscript:

AI	Artificial Intelligence
AMD	Age-related Macular Degeneration
CMS	Center for Medicare and Medicaid Services
CONAN	Consensus Nomenclature for Reporting Neovascular AMD Data
CPT	Current Procedural Terminology
CSCR	Central Serous Chorioretinopathy
CST	Central Subfield Thickness
DME	Diabetic Macular Edema
DRCR	Diabetic Retinopathy Clinical Research
FA	Fluorescein Angiography
FDA	Food and Drug Administration
FSH	ForeseeHome
HRS	Hypo Reflective Spaces
IDTF	Independent Diagnostics Testing Facility
MCPT	Macular Computerized Psychophysical Test
NOA	Notal OCT Analyzer
OCT	Optical Coherence Tomography
PK/PD	Pharmacokinetic/pharmacodynamic
PHP	Preferential Hyperacuity Perimetry
RCV	Reference Change Value
ROC	Receiver Operating Characteristics
SD	Spectral Domain
SNR	Signal to Noise Ratio
T&E	Treat and Extend
VEGF	Vascular Endothelial Growth Factors
